# Successful surgical treatment for recurrent cardiac fibroma 21 years after resection

**DOI:** 10.1186/s40792-015-0043-3

**Published:** 2015-05-16

**Authors:** Kunio Kusajima, Hiroki Hata, Tomoyuki Fujita, Yusuke Shimahara, Shunsuke Sato, Hatsue Ishibashi-Ueda, Junjiro Kobayashi

**Affiliations:** Department of Cardiovascular Surgery, National Cerebral and Cardiovascular Center, Suita, Japan; Department of Pathology, National Cerebral and Cardiovascular Center, Suita, Japan

**Keywords:** Cardiac tumor, Cardiac fibroma, Recurrence, Arrhythmia, Surgery

## Abstract

Cardiac fibromas are rare benign tumors usually seen in the pediatric population. Generally, long-term survival after surgical resection is favorable, and recurrence of fibroma has been hardly reported. Herein, we report a case of a 34-year-old woman who presented with ventricular tachycardia 21 years after resection of a cardiac fibroma and was found to have a recurrent giant cardiac fibroma. We performed a complete resection of the recurrent fibroma. At the 2-year follow-up, she remains asymptomatic with no evidence of ventricular tachycardia or recurrence of fibroma.

## Background

Cardiac fibromas are rare benign tumors predominantly seen in the pediatric population [1]. Patients can be asymptomatic or present with palpitations, cardiac murmur, syncope, arrhythmias, symptoms of congestive heart failure, or sudden death. The clinical presentation depends on tumor location and size [2–5].

Because cardiac fibromas rarely regress spontaneously [6], surgical resection should be considered in symptomatic cases. If possible, complete resection is usually recommended; otherwise, partial resection, heart transplantation, or conservative management is also advocated [3]. Generally, long-term survival after surgical resection is favorable and recurrence has been scarcely reported [3, 5, 6]. In this report, we describe a case of successful resection of a recurrent giant fibroma. To the best of our knowledge, this is the first report of successful surgical treatment of a recurrent cardiac fibroma.

## Case presentation

A 34-year-old woman complained of palpitations and consulted a doctor. She had a history of resection of a right ventricular (RV) fibroma 21 years ago at another hospital. At the initial operation, complete resection was impossible because of its difficulty. Five years after the operation, follow-up was ceased because she did not have any cardiac symptoms.

**Fig. 1:**
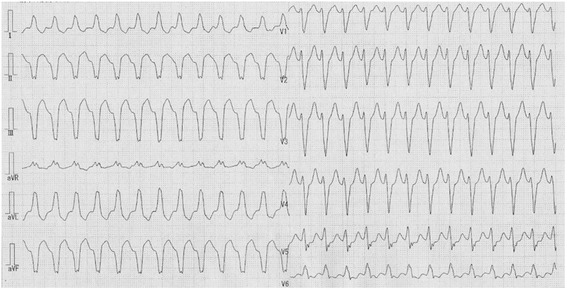
Electrocardiogram at admission showed ventricular tachycardia

**Fig. 2:**
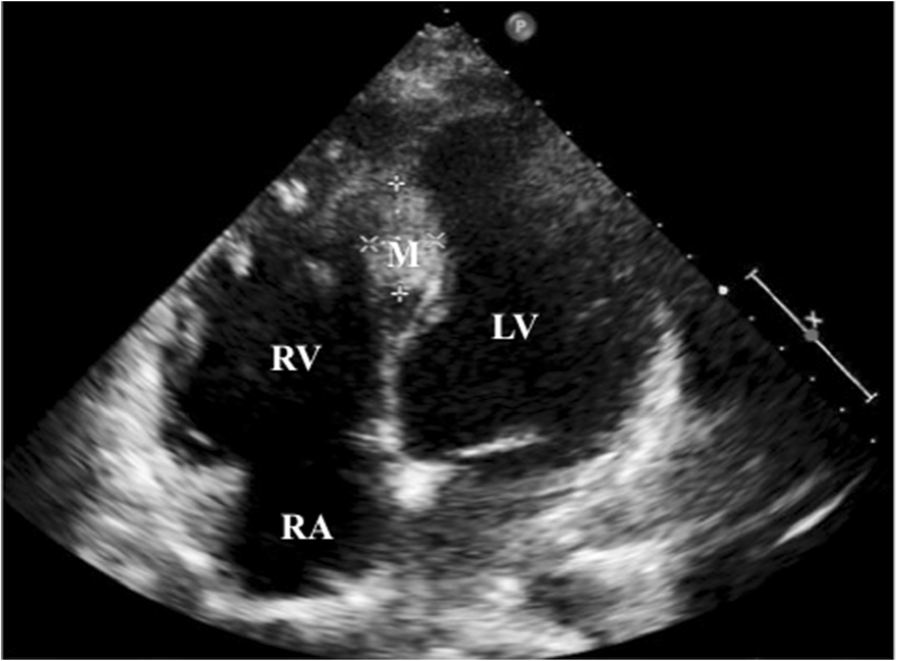
Transthoracic echocardiography image showing a mass in the intraventricular septum. *RA* right atrium, *RV* right ventricle, *LV* left ventricle, *M* mass

**Fig. 3:**
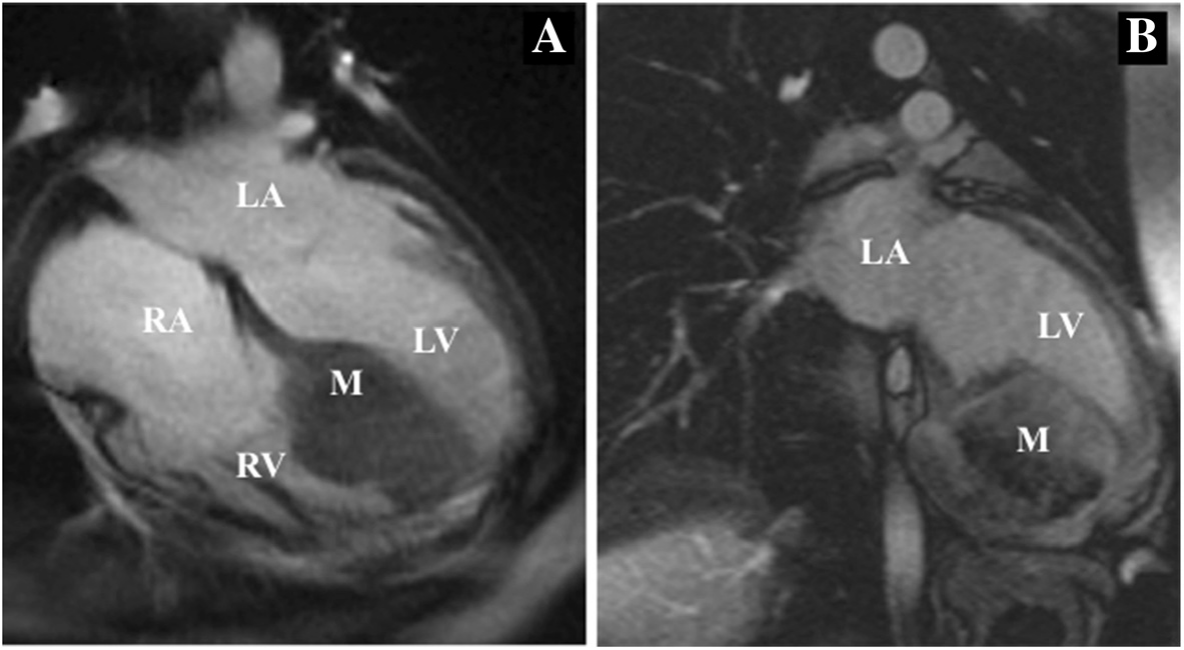
Cardiac magnetic resonance images. **a** A low-density mass located in the intraventricular septum on the T2-weighted image. **b** The mass involved the apex and the posterior wall of the left ventricle (LV) and oppressed the LV cavity. *RA* right atrium, *RV* right ventricle, *LA* left atrium, *LV* left ventricle, *M* mass

When she was referred to our center, electrocardiography showed sustained ventricular tachycardia (VT) (Fig. 1), which was well suppressed with oral amiodarone. Transthoracic echocardiography showed a giant mass (80 × 80 × 60 mm) located in the intraventricular septum (IVS) (Fig. 2) and the posterior wall of the left ventricle (LV). General ventricular wall motion was good (LV ejection fraction of 60%). Tricuspid regurgitation (TR) and mitral regurgitation (MR) were trivial. Enhanced cardiac computed tomography scan revealed that the mass contained calcification and was poorly enhanced. Cardiac magnetic resonance imaging showed that the mass oppressed the LV cavity with low tumor signal on the T2-weighted image (Fig. 3). Positron emission tomography with fluorine-18 fluorodeoxyglucose showed no abnormal uptake within the tumor. These findings strongly suggested the diagnosis of recurrent fibroma. The tumor was huge, oppressing the LV cavity, and was regarded to be the cause of VT. Therefore, we arranged surgical resection.

**Fig. 4:**
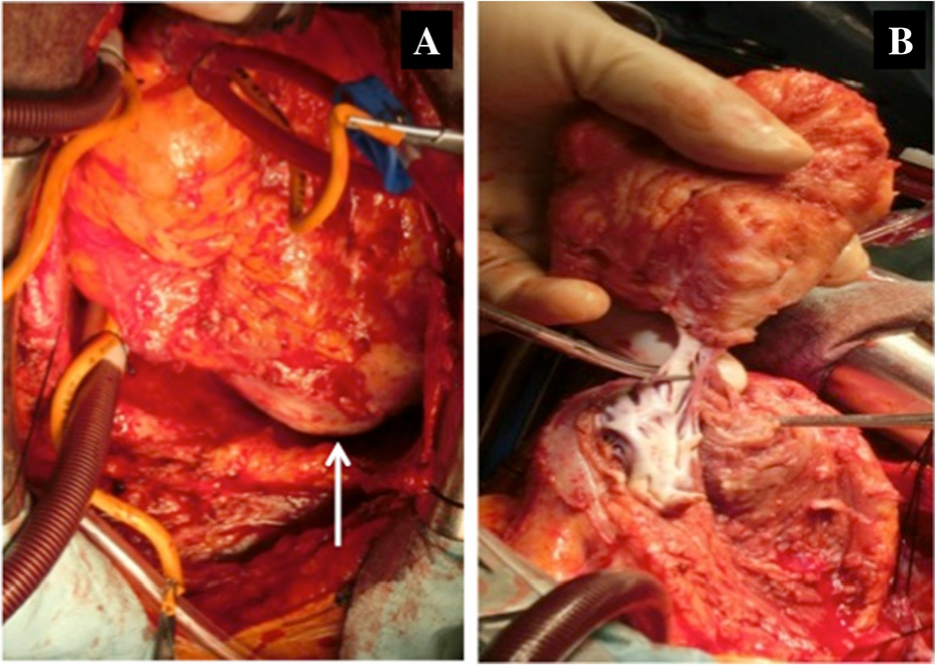
**a** Intraoperative view (patient’s head is at the top). The gray-white tumor located in the apex and the LV posterior wall (*arrow*). **b** The solid and solitary tumor was completely excised by sharp dissection

**Fig. 5:**
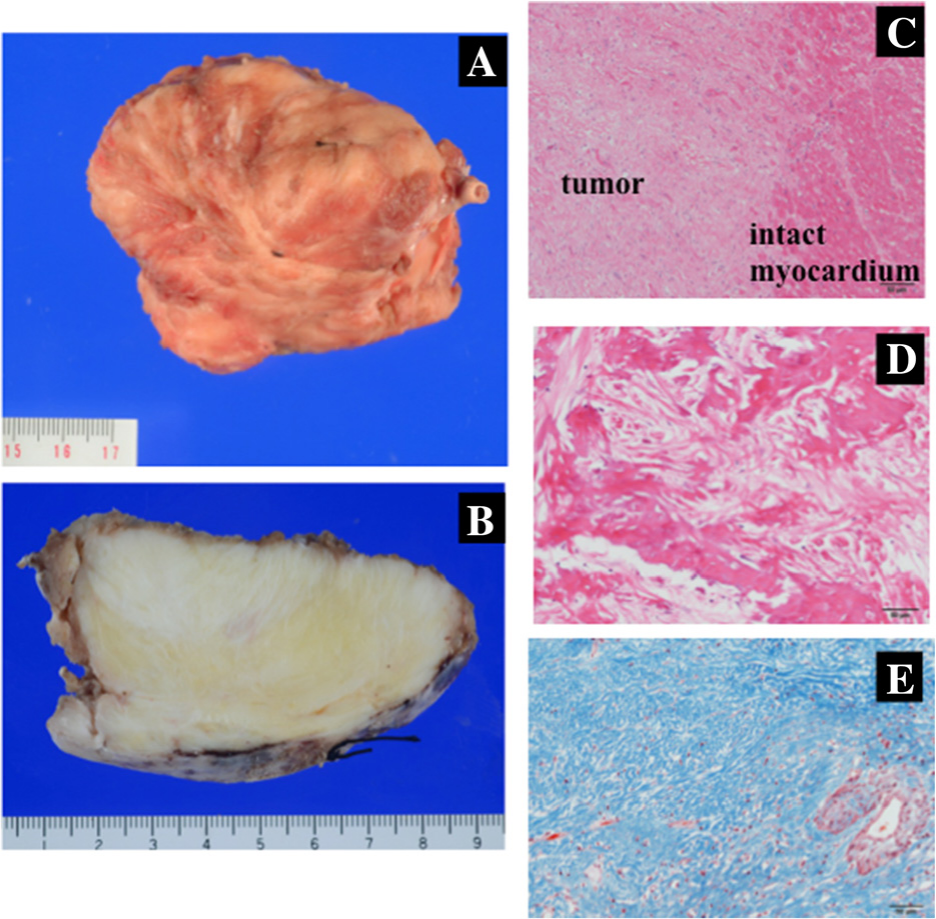
Macroscopic and microscopic appearance of resected fibroma. **a** Resected tumor shows solid, firm, and rounded mass, 80 × 80 × 60 mm in size. **b** Cut surface was whitish yellow and whorled. **c** Border between fibroma and left ventricular myocardium. Hematoxylin and eosin stain ×10. **d** Calcification focus of the tumor, hematoxylin and eosin stain ×20. **e** Tumor composes of fibrous tissue with feeder artery, Masson’s trichrome stain ×20

Complete surgical resection was performed through median sternotomy with cardiopulmonary bypass under cardioplegic arrest. The gray-white tumor appeared to originate from the IVS involving the apex and the LV posterior wall. The solitary tumor was completely excised by sharp dissection together with the feeding artery (Fig. 4). The defect of the IVS, sized 5 × 15 mm, was closed with Teflon felt-pledgeted 4–0 polypropylene mattress sutures. The giant remnant cavity and ventricle wall were closed with double layers of 4–0 polypropylene running sutures. Cardiopulmonary bypass time and aortic cross clamp time were 113 and 64 min, respectively. The patient was easily weaned from cardiopulmonary bypass, and intraoperative transesophageal echocardiography showed good ventricular contraction without TR, MR, or shunt flow through the IVS. The excised specimen showed a well-circumscribed solid firm mass without capsule and measured 80 × 80 × 60 mm, weighing 140 g. Most part of the tumor composed of hyalinized acellular fibrous tissue with focal calcification. Sporadic part showed fibroblast proliferation without malignancy. Pathologic examination confirmed the diagnosis of benign cardiac fibroma (Fig. 5) and total extirpation. Ventricular tachycardia was not observed on continuous electrocardiographic monitoring. Two years after the operation, the patient could cease amiodarone and remained asymptomatic without VT. Transthoracic echocardiography showed good ventricular contraction without residual shunt flow or recurrence of fibroma.

## Discussion

Cardiac fibromas are rare but benign tumors and more than 80% of them occur in the pediatric population [2, 7]. Long-term results after surgical resection were generally reported to be favorable [1, 3]. Therefore, medical follow-up is often ceased once the patient becomes an adult. There has never been a report of a recurrent case after surgical resection to date [3]. In the present case, complete resection was not performed at the initial operation because of diffuse involvement of the fibroma into the right ventricular trabeculae. At this time of operation, the tumor appeared to originate from the right ventricular septum, involving the apex and the posterior wall of LV. Therefore, we believed that this tumor is a recurrent fibroma proliferating from the residual lesion.

The majority of fibromas are found in the LV or RV wall, and fewer than 20% are detected in the IVS [5]. Fibromas can cause lethal arrhythmia, valve regurgitation, and ventricular inflow/outflow tract obstruction depending on their location and size [4, 8]. If left untreated, fibromas can increase the risk of fatality. Therefore, surgical resection is recommended, especially in symptomatic cases. Basically complete resection is recommended first. But, partial resection can be an acceptable option when papillary muscle, valvular, and/or ventricular function might be reduced after complete resection [3, 7].

## Conclusion

In conclusion, we report the first case of a successful complete resection of a recurrent cardiac fibroma 21 years after the initial operation. Recurrence should be considered when new-onset arrhythmia or cardiac murmur is noted in a patient with a history of fibroma resection, especially without complete resection. Although partial resection may be acceptable to preserve valvular and/or ventricular function, complete resection is preferable to eliminate the risk of recurrence.

## Consent

Written informed consent was obtained from the patient for publication of this case report and any accompanying images. A copy of the written consent is available for review by the Editor-in-Chief of this journal.
